# Optimization of a sequential extraction procedure for trace elements in Arctic PM_10_

**DOI:** 10.1007/s00216-020-02874-4

**Published:** 2020-08-20

**Authors:** Eleonora Conca, Mery Malandrino, Agnese Giacomino, Emanuele Costa, Francisco Ardini, Paolo Inaudi, Ornella Abollino

**Affiliations:** 1 Department of Chemistry, University of Turin, Via P. Giuria 5, 10125 Turin, Italy; 2 Department of Drug Science and Technology, University of Turin, Via P. Giuria 9, 10125 Turin, Italy; 3 Department of Earth Sciences, University of Turin, Via V. Caluso 35, 10125 Turin, Italy; 4 Department of Chemistry and Industrial Chemistry, University of Genoa, Via Dodecaneso 31, 16146 Genoa, Italy

**Keywords:** Aerosol particulate matter, Sequential extraction, Trace elements, Source identification

## Abstract

In this work, a two-step sequential extraction scheme for the determination of trace elements in Arctic PM_10_ samples was optimized by using two certified reference materials (CRMs). By means of an experimental design for qualitative variables, the five most common extracting solutions for particulate matter (PM) sequential extraction (high purity water (HPW), 0.032 M HNO_3_, 0.022 M HCl, 0.11 M CH_3_COOH, and 0.012 M CH_3_COOH/CH_3_COONH_4_ buffer) and two different extraction methods (stirring and ultrasounds) were compared. The purpose of the study was the identification of the procedure which gives the best estimation of the anthropogenic portion of the elements present in PM_10_ samples. The use of ultrasounds instead of stirring induced a low but significant decrease of the extraction of all the elements and a decrease in the repeatability of the procedure. Diluted HNO_3_ was the extractant which allowed to maximize the extraction of anthropogenic elements (As, Cd, Pb, Zn) with respect to crustal ones (Al, Si, Ti). The optimized procedure proved successful in avoiding contaminations and, therefore, suitable to be applied to PM samples having extremely low concentrations, such as samples collected in polar or other remote areas. The chosen procedure was applied to ten Arctic PM_10_ samples, allowing for a better identification of their sources. Indeed, it was possible to hypothesize that even though the concentrations of As, Cd, K, Mg, Mn, and Ni in spring and summer were different, their mobility and, therefore, their chemical form in the analyzed PM_10_ samples were probably similar.

**Electronic supplementary material:**

The online version of this article (10.1007/s00216-020-02874-4) contains supplementary material, which is available to authorized users.

**Figure:**
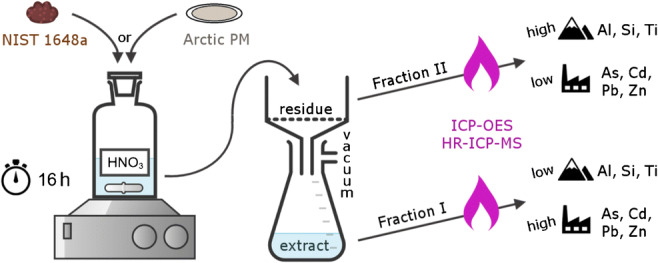
Graphical abstract

## Introduction

Most of the studies on the trace element content of Arctic aerosol particulate matter (PM) consist of the determination of the total concentrations, without distinguishing between the various species, i.e., the different chemical forms of each element [1–4]. Indeed, the small quantity of sample typically collected in the Arctic, together with the chemical complexity of PM and the extremely small particle sizes, can pose significant problems for element speciation [5]. In many cases, the exact distribution of an element between well-defined chemical species is impossible to determine, due to the large number of individual species and to the lack of a universal analytical technique able to both identify and quantify all of them. Alternatively, sequential extraction procedures are often applied, and the classification of analytes is made according to their physical (e.g., size, solubility) or chemical (e.g., bonding, reactivity) properties [5–11]. Apart from the evaluation of the health and environmental impact of PM [7, 12–14], this kind of classification can also be a valid tool for the identification of emission sources [15–17]. Even though speciation obtained by sequential extraction has an operational nature and sometimes lacks selectivity [9], it is possible—in principle—to estimate the anthropogenic portion of the elements present in the sample [18]. Indeed, the anthropogenic portion of the elements in PM is mainly water or acid soluble [19].

Many researchers have worked on the design of extraction schemes for the sequential solubilization of metals from sediments, leading to the development of two commonly accepted sequential extraction procedures: the five-step Tessier protocol [20] and the three-step procedure proposed by the Community Bureau of Reference (BCR) [21]. The latter protocol has the advantage of being harmonized and standardized, and the certified reference material (CRM) BCR 701 (“lake sediment”) is available; on the other hand, the former gives more information about the metal fractions bound to different phases of the sample [22]. Different studies about the application of these extracting schemes or their successive adaptions to PM samples were published, stating that the most interesting and representative fractions are the mobile and the residual ones [23, 24]. As a consequence, many researchers started to develop extraction schemes providing only two fractions, considered a good compromise between costs, analytical times, and resulting data [8, 15–17, 24–34]. Moreover, when dealing with Arctic PM, the application of multi-steps sequential extraction schemes increases the risk of obtaining results below the detection limits, and two-step schemes should be preferred.

Different extracting solutions, different extraction methods, and different techniques for the separation of the extract from the solid residue are currently used for this purpose, thus reducing the possibility to compare results between different studies [13, 24, 28, 32, 35].

The most common extracting solution for PM sequential extraction is deionized or ultrapure water, due to its high representativeness of the natural solubility processes taking place in the environment [16, 19, 22, 24, 30–39]. Nevertheless, water extraction is affected by spontaneous pH changes of the solution, which in turn are influenced by the concentrations of ammonium sulfate and nitrate (secondary species) in PM. For source identification studies, this fact is not desirable, since the results of PM sequential extraction should be directly related to the emission sources and not governed by external factors [8].

Diluted acids are other commonly used extractants, generally allowing a good control of the external factors that could influence the extraction, such as adsorption phenomena on particle surface, complexes formation with organic and inorganic species, and solubility equilibria changes of salts and hydroxides toward acids and bases present in the matrix [13, 15, 19, 35, 36, 40, 41]. The pH generally ranges from 1 to 3, determining the dissolution of sulfates and carbonates but not of iron oxides and silicates [13]. Both weak and strong acids are used, but 0.11 M acetic acid is the most frequent, due to its use in the BCR sequential extraction protocol. Their main drawbacks are the strong overestimation of bioavailability and mobility, and the low selectivity with respect to emission sources [8].

A good alternative of extracting solution for source identification studies is represented by buffers since they are able to maintain a good pH control throughout the extraction. The selected pH is usually very similar to the PM spontaneous one, in order to mimic the interactions naturally occurring in the environment [25, 29].

In the attempt to imitate the conditions of PM interactions with biological systems, many different biological fluids or complexing solutions have also been used [7, 13], but they are not further considered in the present work due to their irrelevance for environmental studies.

Regarding extraction methods, the BCR sequential extraction protocol provides 16 h of stirring. However, in the attempt to reduce the extraction times and/or to enhance the power of the extracting solutions, many alternatives have been tested in the recent years, including the use of ultrasounds (US) [13, 16, 31, 33, 34, 42] or microwaves [24].

As for the separation of the extract from the solid residue, most extraction schemes rely on centrifugation, especially when more than two fractions are involved [6, 22, 37–40]. In two-step extraction schemes, filtration is also quite common, as the filter may subsequently be digested for obtaining the second fraction [26, 34]; nevertheless, a wide variety of filters and filtration systems exist, thus further reducing the comparability of results. Alternatively, some researchers prefer non-sequential extraction schemes, realized by cutting the filter containing the PM sample in two or more parts, from which they obtain the two or more desired fractions [13, 15, 24, 33].

In this study, the most common extracting solutions and extraction methods for PM sequential extraction were compared, in order to optimize a two-step sequential extraction scheme for the operationally valid identification of different chemical forms of trace elements in PM_10_ samples and lay the foundation for a future harmonization of the procedures. The objective of the study was the identification of the procedure which gives the best estimation of the anthropogenic portion of the elements present in PM_10_ samples or, in other words, to identify the procedure which allows to maximize the extraction of elements mainly having an anthropogenic origin and to minimize the extraction of elements mainly having a crustal origin. The CRM NIST 1648a (“urban particulate matter”) and BCR 701 (“lake sediment”) were used for the purpose. A wide variety of elements were analyzed, i.e., Al, As, Ba, Ca, Cd, Co, Cr, Cu, Fe, K, Mg, Mn, Na, Ni, Pb, Sb, Si, Ti, V, and Zn.

In order to evaluate the source identification potential of the optimized procedure, the chosen sequential extraction scheme was applied to ten samples collected in Ny-Ålesund (Svalbard Islands, Norwegian Arctic) in 2010 and 2012, i.e., five samples collected in spring and five samples collected in summer. The choice was made according to previous studies [1, 3], in which these samples appeared the most strongly affected by mid-latitude pollution sources and by local ship emissions, respectively.

## Experimental

### Apparatus and reagents

A Milestone ETHOS One microwave laboratory unit was used for the dissolution of the solid residues. The analysis of extracts and mineralized residuals were carried out using a high-resolution inductively coupled plasma mass spectrometer (HR-ICP-MS) and a inductively coupled plasma optical emission spectrometer (ICP-OES), depending on the concentration levels; model and features of the instruments are reported in Table 1, while operating conditions are reported in Table S1 (see Electronic Supplementary Material, ESM). The reagents used were of analytical purity. Nitric acid, hydrochloric acid, acetic acid, and ammonia were further purified by sub-boiling (s.b.) distillation in a quartz apparatus, from 65%, 37%, 99.8%, and 35% analytical grade solutions respectively. For the digestion of samples, ultrapure (u.p.) hydrogen peroxide (30%) and s.b. nitric acid were used. Water was purified in a Milli-Q system, resulting in high purity water (HPW) with a resistivity of 18 MΩ cm. Element standard solutions were prepared from concentrated (1000 mg L^−1^) stock solutions (Sigma-Aldrich TraceCERT).

**Table 1:** Instrumental techniques used for the analysis

Technique	Model	Features	Analytes
ICP-OES	Perkin Elmer Optima 7000 DV	Mira Mist nebulizer, cyclonic spray chamber, dual Échelle monochro-mator, dual CCD detector	Al, Ca, Fe, K, Mg, Na, Pb, Si, Ti, Zn
HR-ICP-MS	Thermo Finnigan Element 2	Conikal nebulizer, cyclonic spray chamber, magnetic and electric sector, SEM detector	As, Ba, Cd, Co, Cr, Cu, Fe, Mn, Ni, Pb, Sb, Ti, V

### Optimization of the procedure

The most common extracting solutions and extraction methods for PM sequential extraction were compared by means of an experimental design for qualitative variables [43]. Five different extractants, i.e., the most commonly found in literature, were tested, namely HPW [16, 19, 22, 24, 30–37, 42, 44, 45], 0.032 M HNO_3_ (pH = 1.5) [44, 46], 0.022 M HCl (pH = 1.7) [35, 36], 0.11 M CH_3_COOH (pH = 3.0) [21, 40, 41, 47, 48], and 0.012 M CH_3_COOH/CH_3_COONH_4_ buffer (pH = 4.5) [8, 17, 25, 26, 28, 29, 48]. Ammonium was chosen as a cation for the buffer due to the possibility of s.b. distillation of ammonia for further purification. For the preparation of the buffer, 6 mL s.b. ammonia, 11 mL s.b. acetic acid, and 33 mL HPW were used; the solution thus obtained was then diluted 3:1000 prior to be used. The procedure consisted in the introduction of 10 mg of CRM NIST 1648a and 10 mL of the extractant in a 30-mL polycarbonate bottle, which was then either stirred for 16 h at 200 rpm, as required by the BCR protocol [21, 47], or sonicated for 15 min at 500 kW [8, 44]. Afterwards, the suspension was vacuum-filtered through Advantec mixed cellulose ester filter membranes. The solution (fraction I) was then acidified with 100 μL s.b. HNO_3_, and HPW was added up to 20 mL. The filter membrane, containing the insoluble fraction of the sample, was subsequently cut into four pieces with stainless steel scissors and microwave-digested using the vessel-inside-vessel technology [49]: the 30-mL tetrafluoromethoxyl (TFM) vessels containing the samples and the digestion mixture were inserted into 100-mL TFM vessels. According to the current legislation of the European Community in the field of air quality monitoring (UNI EN 14902:2005), the digestion mixture was composed of 2 mL s.b. HNO_3_ and 0.5 mL u.p. H_2_O_2_; a mixture of 10 mL HPW and 1 mL H_2_O_2_ was introduced in the bigger vessel and the temperature was ramped up to 220 °C within 20 min, followed by a dwell time of another 20 min. The obtained solution (fraction II) was subsequently filtered through a Whatman Grade 5 cellulose filter (porosity 2.5 μm) to remove any insoluble particles, and HPW was added up to 20 mL. All the possible steps of the sequential extraction procedure were performed in a clean environment under a Class-100 laminar flow bench-hood, to avoid any possible contamination. Considering that no HF was used for the digestion, silicates and other refractory compounds were not solubilized by the digestion mixture. Therefore, extraction percentages were calculated with respect to the sum of fraction I and fraction II. Each extraction procedure was performed at least in triplicate. Moreover, an additional repetition of each test was performed for analyzing the residue by means of a scanning electron microscopy-energy dispersive X-ray spectrometer (SEM-EDS); in this case, a quartz filter (Pallflex Tissuquartz) was used to filter the solution. All the possible combinations of the five extracting solutions and the two extraction methods were tested; therefore, a total of ten procedures was applied.

The five studied extractants were also tested on the CRM BCR 701, by applying 16-h stirring as the only extraction method. Exclusively, the six certified elements, namely Cd, Cr, Cu, Ni, Pb, and Zn, were determined in the BCR 701 extracts. In this case, extraction percentages were calculated with respect to the certified total content, in order to be able to compare our results with the certified fractions.

### Test of the procedure on Arctic PM_10_ samples

In order to evaluate the source identification potential of the optimized procedure, the chosen sequential extraction protocol was applied to ten samples collected in Ny-Ålesund (Svalbard Islands, Norwegian Arctic) in 2010 and 2012. During the 2010 and 2012 sampling campaigns, PM_10_ samples were collected from 16th March to 16th September 2010 and from 17th April to 7th September 2012, with a 4-day resolution. For this work, five samples collected in spring (namely 23Mar10, 27Mar10, 17Apr12, 25Apr12, and 29Apr12) and five samples collected in summer (namely 26Jun10, 9Jul10, 18Jul12, 22Jul12, and 26Jul12) were chosen. The sample name corresponds to the sampling start date. The choice was made according to previous studies [1, 3], in which these samples appeared the most strongly affected by mid-latitude pollution sources and by local ship emissions, respectively. Even though an anthropogenic contribution was identified in the selected samples, the concentrations were quite low, in line with previous works on Arctic and Antarctic PM_10_ samples [1, 3, 50].

The sampling was performed by means of PTFE hydrophilic filters (Advantec, product code: H100A090C, 90-mm diameter, efficiency > 99% for 0.3-μm particles) and an Echo HiVol sampler (TCR Tecora, 200 L/min). After each sampling, filters were placed in polycarbonate Petri dishes, sealed and immediately frozen; samples were maintained at − 20 °C during all stages of transportation to Italy and storage. More information on the sampling location and strategy can be found elsewhere [1, 3, 51, 52].

The application of the sequential extraction procedure to the PM_10_ samples collected in Ny-Ålesund was performed exactly as explained in the “Optimization of the procedure” in the “Experimental” section for the CRM powder. However, in this case, one-half of each filter was cut into small pieces with stainless steel scissors and immediately introduced into the polycarbonate bottle. After the extraction, the suspension containing the insoluble fraction of the sample and the pieces of the PTFE filter was vacuum-filtered. The filter membrane was subsequently cut into four pieces with stainless steel scissors and microwave-digested, together with the pieces of the sampling PTFE filter. Extraction percentages were calculated with respect to the sum of fraction I and fraction II.

Procedural blanks were prepared similarly to the samples, by applying the sequential extraction procedure to three blank filter halves which had undergone the transport to and from the sampling site; procedural blank concentrations (PB) were subtracted from sample concentrations, in order to eliminate the filter, travel, and storage contributions.

### Statistic data analysis

The experimental design (or design of experiments—DOE) is a mathematical modeling approach widely used in chemistry for the optimization of processes or procedures [53–56]. A response surface, in which both single and interaction effects of the variables of interest are taken into account, is generally built by means of a regression analysis. An experimental design for qualitative (categorical) variables [43] was performed by means of the R-based software CAT (Chemometric Agile Tool) [57]. The investigated variables were the extractant and the extraction method, as explained in the “Optimization of the procedure” in the “Experimental” section. Considering that the purpose of the study was to identify the procedure which allows to maximize the extraction of elements mainly having an anthropogenic origin (hereafter referred to as “anthropogenic elements”) and to minimize the extraction of elements mainly having a crustal origin (hereafter referred to as “crustal elements”), two different responses were alternatively used for computing the multilinear regression model, i.e., the mean of the extraction percentages obtained for some anthropogenic elements (namely As, Cd, Pb, and Zn) and the mean of the extraction percentages obtained for some crustal elements (namely Al, Si, and Ti). The attribution of the main element sources in the CRM NIST 1648a was made according to Marine and Crustal Enrichment Factors (MEFs and CEFs, ESM Table S2), calculated for this CRM with respect to the average sea and upper crust composition [58, 59], by using Na and Al as reference elements, respectively [1].

The Mann-Whitney test was used for verifying if the results of the extractions performed with two alternative procedures or on two sets of samples were significantly different [60, 61]. XlStat 2018.1 software package, an add-on of Microsoft Excel, was used for performing the calculations.

## Results and discussion

### Optimization of the procedure

**Fig. 1:**
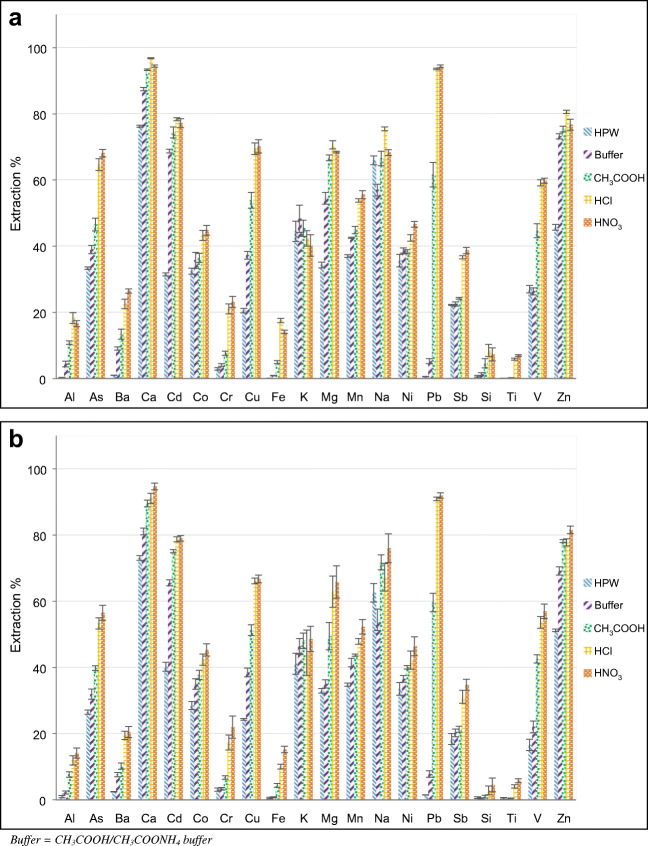
Extraction percentages obtained in fraction I for the CRM NIST 1648a with the five tested extracting solutions: **a** 16-h stirring; **b** 15-min ultrasounds. The error bars represent the standard deviations of the three replicates

Figure 1 shows the extraction percentages obtained in fraction I for the CRM NIST 1648a with the five tested extracting solutions (HPW, buffer, CH_3_COOH, HCl, and HNO_3_) and the two applied extraction methods (16-h stirring and 15-min ultrasounds). Table 2 reports the calculated extraction percentages for fraction I, while Table S3 (see ESM) reports the recovery percentages calculated for the sum of the concentration obtained in fraction I and fraction II, with respect to the certified values. The relative standard deviation (RSD%) was generally lower than 5% and almost always lower than 10%. Occasional higher values were registered for Al, Fe, Pb, Si, and Ti in fraction I and for Ca, K, Na, and Si in fraction II.

**Table 2:** Extraction percentages obtained in fraction I for the CRM NIST 1648a with the ten tested procedures

	HPW—stir	Buffer—stir	CH_3_COOH—stir	HCl—stir	HNO_3_—stir	HPW—US	Buffer—US	CH_3_COOH—US	HCl—US	HNO_3_—US
Al	0.32 ± 0.01	4.4 ± 0.9	10.9 ± 0.6	18 ± 2	16.6 ± 0.9	1.0 ± 0.3	2.2 ± 0.5	7.6 ± 0.8	12 ± 1	14 ± 2
As	33.4 ± 0.4	39 ± 1	47 ± 2	65 ± 2	68 ± 1	26.5 ± 0.7	32 ± 2	39.8 ± 0.7	53 ± 2	57 ± 2
Ba	1.03 ± 0.05	9.1 ± 0.5	13 ± 2	23 ± 1	26.5 ± 0.7	2.4 ± 0.1	7.6 ± 0.7	10.2 ± 0.9	20 ± 1	21 ± 2
Ca	76.3 ± 0.3	87.5 ± 0.5	93.3 ± 0.2	96.8 ± 0.2	94.4 ± 0.3	73.1 ± 0.7	81 ± 1	90 ± 1	91 ± 2	95 ± 1
Cd	31.5 ± 0.5	68.7 ± 0.6	74 ± 2	78.4 ± 0.4	77 ± 1	40 ± 1	65.6 ± 0.9	75.1 ± 0.5	78.7 ± 0.7	79.0 ± 0.8
Co	32.5 ± 0.9	36 ± 2	37 ± 1	43 ± 2	45 ± 2	29 ± 1	35 ± 2	38 ± 1	42 ± 2	45 ± 2
Cr	2.9 ± 0.4	4.1 ± 0.4	7.7 ± 0.7	21 ± 2	23 ± 2	3.1 ± 0.5	3.3 ± 0.4	6.8 ± 0.5	17 ± 2	22 ± 3
Cu	20.5 ± 0.7	37 ± 1	54 ± 2	70 ± 2	70 ± 2	24.3 ± 0.3	39 ± 1	51 ± 2	66.2 ± 0.9	67 ± 1
Fe	0.14 ± 0.01	0.89 ± 0.06	5.0 ± 0.5	17.6 ± 0.6	14.1 ± 0.5	0.5 ± 0.2	0.8 ± 0.1	4.3 ± 0.6	10.0 ± 0.7	15 ± 1
K	45 ± 3	48 ± 4	45 ± 2	42 ± 2	40 ± 3	41 ± 3	46 ± 2	48 ± 2	44 ± 7	49 ± 4
Mg	34.3 ± 0.9	55 ± 2	66.7 ± 0.8	71 ± 1	68.5 ± 0.3	32.9 ± 0.7	35 ± 1	50 ± 4	63 ± 5	66 ± 5
Mn	37.1 ± 0.4	42.6 ± 0.2	45 ± 1	53.8 ± 0.5	56 ± 1	34.8 ± 0.5	42 ± 1	43.7 ± 0.4	47.8 ± 0.9	52 ± 2
Na	66 ± 1	57 ± 2	67 ± 2	75.5 ± 0.6	68.3 ± 0.8	63 ± 3	54 ± 3	72 ± 2	67 ± 4	76 ± 4
Ni	36 ± 2	38.7 ± 0.8	38.3 ± 0.6	43 ± 1	46.6 ± 0.9	34 ± 2	37 ± 1	40.0 ± 0.5	42 ± 3	46 ± 3
Pb	0.57 ± 0.07	5.3 ± 0.8	62 ± 4	93.5 ± 0.2	94.3 ± 0.4	1.5 ± 0.1	8 ± 1	60 ± 3	90.9 ± 0.5	92.0 ± 0.8
Sb	22.3 ± 0.2	22.6 ± 0.7	24.3 ± 0.3	36.7 ± 0.5	38.7 ± 0.9	18 ± 2	20 ± 1	21.3 ± 0.9	31 ± 2	35 ± 2
Si	0.73 ± 0.3	1.3 ± 0.5	5 ± 1	9 ± 2	7 ± 2	0.7 ± 0.3	0.4 ± 0.2	1.0 ± 0.4	3 ± 1	5 ± 2
Ti	0.08 ± 0.04	0.07 ± 0.02	0.22 ± 0.04	5.9 ± 0.3	7.0 ± 0.3	0.5 ± 0.3	0.2 ± 0.1	0.50 ± 0.01	4.1 ± 0.5	5.8 ± 0.6
V	27 ± 1	26 ± 1	45 ± 2	59.2 ± 0.9	59.8 ± 0.8	17 ± 2	22 ± 2	43 ± 1	54 ± 2	57 ± 2
Zn	45.7 ± 0.9	73.2 ± 0.8	75.4 ± 0.9	80.6 ± 0.5	77 ± 2	51.2 ± 0.4	69 ± 1	78.2 ± 0.4	78 ± 1	82 ± 1

Stir = 16-h stirring; US = 15-min ultrasounds; buffer = CH_3_COOH/CH_3_COONH_4_ buffer

Regarding extraction percentages obtained in fraction I, most of the analyzed alkaline and alkali-earth metals (namely Ca, K, Mg, and Na), commonly deriving from marine and—to a lesser extent—crustal sources, showed extraction percentages ranging from 33 to 100%; the elements typically having a crustal origin (i.e., Al, Si, and Ti) are generally poorly dissolved and, accordingly, their extraction percentages ranged from 0.071 to 18%; the elements often having a mixed crustal-anthropogenic origin (i.e., Ba, Co, Fe, Mn, Ni, and V) had extraction percentages ranging from 0.14 to 60%; the extraction percentages obtained for most of the elements typically having a prevalent anthropogenic origin (i.e., As, Cd, Cu, and Zn) were considerably higher, ranging from 21 to 82%; Cr, Pb, and Sb, which are typically anthropogenic, gave low extraction percentages, due to their specific chemistry: Cr (2.9–23%) and Sb (18–39%) can be completely extracted only by concentrated HF and HCl [18, 62], respectively, while the extraction of Pb (0.57–94%) was strongly affected by the extractant pH. With exception to alkaline metals (i.e., K and Na), the extraction percentages significantly increased when the pH of the extractant decreased. In particular, when stirring was used as the extraction method, the extraction percentages registered for Pb were 0.57%, 5.3%, 62%, and 94% for HPW, buffer (pH 4.5), CH_3_COOH (pH 3.0), and HCl or HNO_3_ (pH 1.5–1.7), respectively. Other elements that show very different extraction percentages according to the extractant used included As, Cd, Cu, Mg, V, and Zn; most of them often derive from anthropogenic sources. In addition, the extraction of the elements generally having a crustal or a mixed crustal-anthropogenic origin was also influenced by variations in the extractant pH.

This behavior underlines the necessity of harmonizing the PM sequential extraction procedures, as the results obtained with different procedures are undoubtedly not comparable and, hence, not helpful for the identification of the element sources.

A multilinear regression model was computed for evaluating how the choice of the extractant and the extraction method influenced the extraction of anthropogenic elements. The resulting model is the following:

MOD1 $$ y=28.1+49.3\ {e}_1+48.4\ {e}_2+34.9\ {e}_3+16.2\ {e}_4+1.6\ m $$

where *e*_*1*_*-e*_*4*_ represent the usage of HNO_3_, HCl, CH_3_COOH, and CH_3_COOH/CH_3_COONH_4_ buffer as extractant, respectively, and *m* represents the usage of 16-h stirring as extraction method. For each factor, the reference level (HPW as extractant and US as extraction method) was the lowest, i.e., the one causing the smallest extraction of the selected anthropogenic elements. All the factors resulted significant (*p* < 0.001).

Similarly, a multilinear regression model was computed for evaluating how the choice of the extractant and the extraction method influenced the extraction of crustal elements. The resulting model is the following:

MOD2 $$ y=\left(-0.3\right)+8.5\ {e}_1+7.9\ {e}_2+3.5\ {e}_3\ \left(+0.8\ {e}_4\right)+1.9\ m $$

where the coefficients in brackets resulted not significant (*p* > 0.05), while all the others were significant (*p* < 0.001). For each factor, again, the reference level (HPW as extractant and US as extraction method) was the lowest, i.e., the one causing the smallest extraction of the selected crustal elements. Nevertheless, the difference between the extracting power of HPW and the extracting power of the buffer (*e*_*4*_) was not significant for these analytes.

The use of 15-min ultrasounds instead of the typical 16-h stirring induced a low but significant (*p* < 0.001) decrease of the extraction of both anthropogenic and crustal elements. This result was confirmed by the composition of the residues analyzed by SEM-EDS as, for most of the analytes (including non-metals such as Cl, P, and S), the concentration was higher when ultrasounds were used instead of stirring. In addition, the Mann-Whitney test demonstrated that the use of stirring gave more reproducible results than ultrasounds, as the relative standard deviations of the latter procedure were significantly higher (*p* < 0.05) for many analytes, namely Al, Ca, Cr, Mg, Na, Sb, and Si. Therefore, the application of 16-h stirring should be preferred.

Even though most of the coefficients of MOD2 were lower than the coefficients of MOD1, all the signs were positive; therefore, it was not possible to identify a procedure able to maximize the extraction of anthropogenic analytes while minimizing the extraction of crustal ones. Nevertheless, by subtracting the coefficients of MOD2 from the ones computed in MOD1, it was possible to obtain a measure of the higher extractability of anthropogenic elements with respect to crustal ones:

MOD3 $$ y=28.4+40.8\ {e}_1+40.5\ {e}_2+31.4\ {e}_3+17.0\ {e}_4-0.3\ m $$

It is evident that even though anthropogenic elements are always more easily extractable than crustal elements, this difference is dependent on the extractant pH and is the highest for HNO_3_ (*e*_*1*_). This means that even though the use of a low-pH extractant caused an increase of the extraction percentages of both crustal and anthropogenic elements, the latter showed a more marked increase.

**Fig. 2:**
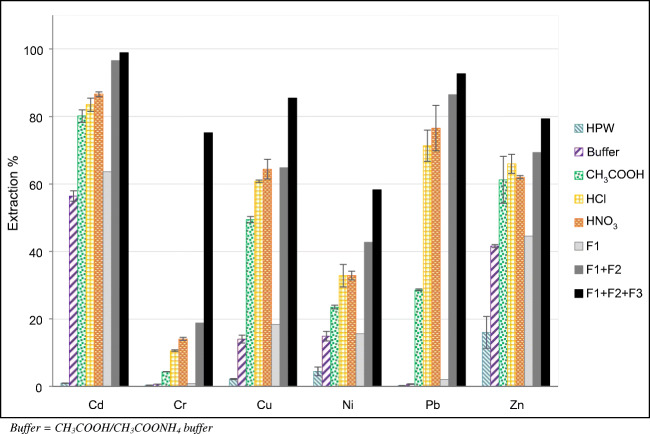
Extraction percentages obtained in fraction I for the CRM BCR 701 with the five tested extracting solutions and 16-h stirring; for comparison, the certified extraction percentages for the BCR sequential extraction protocol are also reported (F1 = fraction 1; F1 + F2 = fraction 1 + fraction 2; F1 + F2 + F3 = fraction 1 + fraction 2 + fraction 3). The error bars represent the standard deviations of the three replicates

In order to clearly identify the extractant representing the best compromise, we critically compared these results with the ones obtained for the CRM BCR 701. Figure 2 shows the extraction percentages obtained in fraction I with the five tested extracting solutions; considering the results obtained for the CRM NIST 1648a, 16-h stirring was the only extraction method used. For comparison, the certified extraction percentages related to the BCR sequential extraction protocol are also reported in Fig. 2. Table S3 (see ESM) reports the calculated extraction percentages for both fraction I and fraction II.

According to the general thought that the first fraction of the BCR protocol represents a good estimate of the anthropogenic portion of each metal [18, 19], it is possible to state that water gave underestimated results. Furthermore, taking into account that HPW extractions are strongly influenced by the pH of the sample [8, 63], the use of this extractant should be avoided.

The extraction percentages obtained with CH_3_COOH/CH_3_COONH_4_ buffer were the nearest to the first certified fraction of the BCR protocol. Nevertheless, only the 5.3% of Pb was extracted when this procedure was applied to the CRM NIST 1648a. Considering that the PM which constitutes the CRM NIST 1648a was collected in an urban area in 1976–1977, when the use of tetraethyllead in gasoline was still widespread, we can hypothesize that the anthropogenic portion of Pb is much higher. Nevertheless, this hydrophobic organometallic form of Pb proved to be not extractable with this buffer. Therefore, this buffer is probably not suitable to discriminate between anthropogenic and crustal forms of Pb.

Similarly, an extraction percentage of 62% for Pb in the CRM NIST 1648a, found by extraction with CH_3_COOH, was likely an underestimation of the anthropogenic portion of Pb in the urban PM collected in those years. Nevertheless, it is interesting to note that the extraction percentages obtained with CH_3_COOH for the CRM BCR 701 were significantly higher than the expected ones (F1 in Fig. 2), even though the applied extractant and extraction method are the same. The reason of this discrepancy lies in the different solid/extractant ratio chosen with respect to the one provided by the BCR protocol: the latter, which is designed to be applied to soil or sediment samples, provides a solid/extractant ratio of 25 g L^−1^ [21, 47], while in this work, we applied a solid/extractant ratio of 1 g L^−1^. Due to the small quantity of PM samples typically collected, the usage of the solid/extractant ratio provided by the BCR protocol would not be achievable. Nevertheless, some elements (e.g., Pb) are extremely sensitive to small changes of this ratio, according to their specific partition coefficient [64, 65]. As suggested by Bacon et al. (2008), it is extremely important to define a constant solid/extractant ratio to be applied to the sequential extraction of PM samples, in order to reduce the operational nature of the results and to be able to compare data obtained by different researchers.

In accordance with their lower pH, the procedures in which diluted HCl and HNO_3_ were used as extractants generally gave the highest extraction percentages, which often reached but did not exceed the sum of the certified first and second fraction (F1 + F2 in Fig. 2). According to the Mann-Whitney test (*p* < 0.05), the extraction percentages obtained with the two acids were not significantly different, probably due to their very similar pH.

Considering the results obtained with the two CRMs, it is possible to state that HPW, CH_3_COOH, and CH_3_COOH/CH_3_COONH_4_ buffer are not appropriate extractants for giving a good estimation of the anthropogenic portion of the elements present in PM samples. HCl and HNO_3_ generally gave very similar results, but it appears that HNO_3_ was the extractant which allowed to maximize the extraction of anthropogenic elements with respect to crustal elements. Moreover, due to the formation of polyatomic interferences (e.g., ArCl and ClO), the use of HCl can cause problems in the ICP-MS determination of some elements (e.g., As and Cr). Consequently, when the sequential extraction procedure is intended for the determination of a wide variety of analytes, the use of HCl should be avoided and substituted with HNO_3_.

For all these reasons, the procedure tested on the ten Arctic PM_10_ samples was the one relying on the use of 0.032 M HNO_3_ as the extractant and 16-h stirring as the extraction method.

### Test of the procedure on Arctic PM_10_ samples

The results of the sequential extraction procedure (ESM Table S5) are expressed as percentages, with respect to the total extraction (i.e., sum of fraction I and fraction II). Except for two results for Cd, three results for Si and three results for Zn, all the determined concentrations were higher than the corresponding procedural blanks (PB). Therefore, due to the absence of a sufficient amount of data, Si and Zn were not included in the following statistical treatments. Despite the long sample manipulation that the procedure requires, the use of a laminar flow hood and of sub-boiling and ultrapure reagents proved successful in avoiding contaminations. Hence, the optimized procedure is suitable to be applied to PM samples having extremely low concentrations, such as samples collected in polar or other remote areas.

**Fig. 3:**
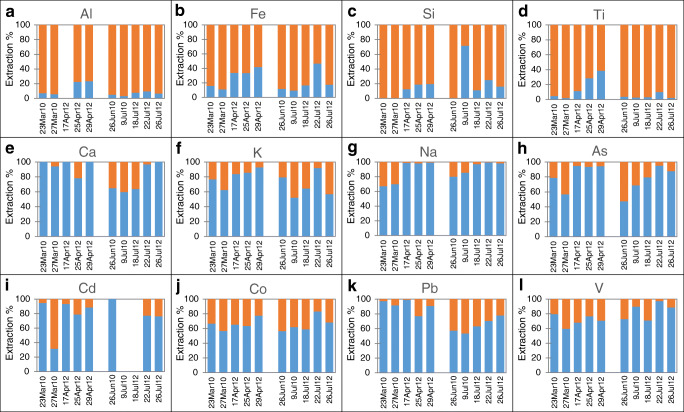
Extraction percentages obtained for the ten Arctic PM_10_ samples for **a**–**d** prevalently crustal elements; **e**–**g** prevalently marine elements; **h**–**l** prevalently anthropogenic elements

The results are generally consistent with the analyte primary sources inferable from the calculated EFs (ESM Table S6). As expected, prevalently geogenic analytes (i.e., Al, Fe, Si, and Ti), generally characterized by low CEFs, were mostly present in fraction II (Fig. 3). Indeed, these elements are often strongly bound to the mineral structure of the particles; therefore, they are not easily extractable. Cr is also mainly present in fraction II (ESM Fig. S1) but, as explained in the “Optimization of the procedure” in the “Results and discussion” section, this is due to its specific chemistry [18]. Conversely, both prevalently marine analytes (i.e., Ca, K, Na), characterized by low MEFs, and prevalently anthropogenic analytes (i.e., As, Cd, Co, Cu, Ni, Pb, V, and Zn), generally characterized by high CEFs and high MEFs, were prevalently present in fraction I (Fig. 3 and ESM Fig. S1). In fact, in PM samples collected in the vicinity of the sea, the former group of analytes is generally present in the form of soluble salts. On the other hand, anthropogenic elements are often weakly bound to atmospheric particles, by means of adsorption processes. Ba and Sb showed extremely variable extraction percentages, with fraction I ranging from 20 to 74% and from 16 to 74%, respectively, generally higher for spring samples than for summer samples (ESM Fig. S1). In addition, Mg and Mn showed extremely variable extraction percentages, remarkably lower for 2010 (12–29% and 19–51%, respectively) than for 2012 samples (85–98% and 50–82%, respectively) (ESM Fig. S1). This different chemical behavior can possibly reflect a variation of the main sources of these elements between the two sampling campaigns.

The Mann-Whitney test was performed on the results of the sequential extraction procedure for checking if significant differences were present between the two groups of samples, i.e., between the five samples collected in spring and the five samples collected in summer. The results obtained for the two groups of samples for As, Ca, Cd, Co, Cr, Cu, Fe, K, Mg, Mn, Na, Ni, Si, and Ti were not significantly different. Considering that the chemical behavior of these elements (i.e., their extractability) in the Arctic PM_10_ samples did not change throughout the sampling campaigns, it is possible to hypothesize that their main sources remained the same all over the spring-summer seasons. On the other hand, significant differences were found for Ba, Pb, Sb, and V (*p* < 0.1) and for Al (*p* < 0.15). All these elements, except V, showed higher extraction percentages in spring samples than in summer samples. This result is coherent with the previous identification of long-range transport processes as the main source of PM_10_ elements in spring, and of cruise ship emissions as the main source of V in summer. The latter is shown by the Ce-La-V ternary diagram of Fig. S2 (see ESM), in which the five samples collected in summer appeared the most strongly influenced by oil combustion sources. Indeed, for building this graph, the concentrations of Ce and La were multiplied by an adequate coefficient (1.54 and 3.1 respectively) so that the center of the diagram represents the composition of the upper crust [66]. In this way, PM samples that are strongly influenced by refinery emissions or by oil combustion processes (e.g., ship emissions) are generally located close to the lower left or right corner of the diagram, respectively, while PM samples strongly influenced by vehicular traffic emissions are likely located close to the triangle center, slightly toward Ce [1, 46, 67, 68].

PCA (ESM Fig. S3) allowed to visualize both the slight difference between the samples collected during the two sampling campaigns (PC1, 51.05% of the total variance—the extraction percentages of 2012 samples were generally higher than the ones obtained for 2010 samples) and the differences between spring and summer samples (PC2, 14.06% of the total variance). Pb and V appeared to be the elements which most strongly determined a differentiation of the two groups, as they were much more easily extractable in spring and summer samples, respectively. This can be explained considering that these elements are typically anthropogenic, with Pb mainly deriving from mid-latitude pollution sources and V often deriving from local ship emissions. In addition, Al, Ba, Ca, Sb, and Ti appeared also slightly more easily extracted in spring samples, while Co, Cr, and Na appeared slightly more easily extracted in summer samples.

By performing the Mann-Whitney test on the concentrations obtained by direct digestion of these ten samples (ESM Table S7 [1, 3]), significant differences between the two groups of samples were found for As, Ba, Mn, Ni, Pb, and V at a significance level of 95% and for Cd, K, and Mg at a significance level of 85%. On the other hand, the results obtained for Al, Ca, Co, Cr, Cu, Fe, Na, Ti, and Zn were not significantly different. No comparison could be made for Sb and Si, since these elements were not determined when the direct digestion of samples was performed. The results of the Mann-Whitney test implied that for Ba, Pb, and V, the sequential extraction procedure confirmed the source identification resulting from the direct digestion of the PM_10_ samples. Moreover, through the sequential extraction procedure, it was possible to distinguish the two groups of samples by means of the concentrations of Al (which in the direct digestion was not significantly different) and not by means of the concentrations of As, Cd, K, Mg, Mn, and Ni (which in the direct digestion were significantly different). This probably means that even though the concentrations of the latter group of analytes in the two investigated periods were different, their mobility and, therefore, their chemical form in the analyzed PM_10_ samples were similar. This can be possibly due to a different contribution of the same source and not to the presence of different sources. The most likely sources for these elements, as detailed in our previous work [1], are continental anthropogenic activities (e.g., incinerators or industries) for As and Cd, marine spray for K and Mg, and occasional local ship emissions for Ni. On the contrary, even though the concentrations of Al in the two investigated periods were similar, the chemical behavior of this analyte and, therefore, its species present in the PM_10_ samples were probably different. This can be possibly due to the presence, in the two seasons, of different crustal sources (e.g., weathering of the upper crust occurred in different geographical areas) [69].

## Conclusions

In this study, the most common extracting solutions and extraction methods for PM sequential extraction were compared, in order to optimize a two-step sequential extraction scheme and lay the foundation for a future harmonization of the procedures. Two CRMs were used for the purpose, i.e., NIST 1648a (“urban particulate matter”) and BCR 701 (“lake sediment”).

The use of 15-min ultrasounds instead of the typical 16-h stirring induced a low but significant decrease of the extraction of both anthropogenic and crustal elements and a decrease in the reproducibility of the procedure; the latter extraction method should therefore be preferred.

As expected, the extraction percentages generally increased when the pH of the extractant decreased. Diluted HNO_3_ (0.032 M) was the extractant which allowed to maximize the release of anthropogenic elements with respect to crustal elements. Moreover, this extractant allows to avoid the unnecessary formation of polyatomic interferences when ICP-MS is chosen as analytical technique.

In order to evaluate the source identification potential of the optimized procedure, the chosen sequential extraction scheme was applied to five samples collected in spring and five samples collected in summer in Ny-Ålesund in 2010 and 2012. The Mann-Whitney test was performed on both the results of the sequential extraction procedure and of direct digestion for checking if significant differences were present between the two groups of samples. For some analytes (namely Ba, Ca, Cu, Fe, Na, Pb, and V), the sequential extraction procedure confirmed the source identification resulting from the direct digestion of the PM_10_ samples. However, for other analytes, the two tests gave different results, suggesting that even though the concentrations of As, Cd, K, Mg, Mn, and Ni in the two investigated periods were different, their mobility and, therefore, their chemical form in the analyzed PM_10_ samples was probably similar.

## Electronic supplementary material

ESM 1 (PDF 469 kb)

## References

[1] Conca E, Abollino O, Giacomino A, Buoso S, Traversi R, Becagli S (2019). Source identification and temporal evolution of trace elements in PM10 collected near to Ny-Ålesund (Norwegian Arctic). Atmos Environ.

[2] Giardi F, Traversi R, Becagli S, Severi M, Caiazzo L, Ancillotti C (2018). Determination of rare earth elements in multi-year high-resolution Arctic aerosol record by double focusing inductively coupled plasma mass spectrometry with desolvation nebulizer inlet system. Sci Total Environ.

[3] Bazzano A, Ardini F, Grotti M, Malandrino M, Giacomino A, Abollino O (2016). Elemental and lead isotopic composition of atmospheric particulate measured in the Arctic region (Ny-Ålesund, Svalbard Islands). Rendiconti Lincei.

[4] Singh DK, Kawamura K, Yanase A, Barrie LA (2017). Distributions of polycyclic aromatic hydrocarbons, aromatic ketones, carboxylic acids, and trace metals in Arctic aerosols: long-range atmospheric transport, photochemical degradation/production at polar sunrise. Environ Sci Technol.

[5] Huggins FE, Huffman GP, Robertson JD (2000). Speciation of elements in NIST particulate matter SRMs 1648 and 1650. J Hazard Mater.

[6] Templeton MD, Ariese F, Cornelis R, Danielsson LG, Muntau H, van Leeuwen HP (2000). Guidelines for terms related to chemical speciation and fractionation of elements. Definitions, structural aspects and methodological approaches. Pure Appl Chem.

[7] Pelfrêne A, Cave MR, Wragg J, Douay F (2017). In vitro investigations of human bioaccessibility from reference materials using simulated lung fluids. Int J Environ Res Public Health.

[8] Canepari S, Astolfi ML, Moretti S, Curini R (2010). Comparison of extracting solutions for elemental fractionation in airborne particulate matter. Talanta..

[9] Bacon JR, Davidson CM (2008). Is there a future for sequential chemical extraction?. Analyst.

[10] Abollino O, Giacomino A, Malandrino M, Mentasti E, Aceto M, Barberis R (2006). Assessment of metal availability in a contaminated soil by sequential extraction. Water Air Soil Pollut.

[11] Casalino CE, Malandrino M, Giacomino A, Abollino O (2013). Total and fractionation metal contents obtained with sequential extraction procedures in a sediment core from Terra Nova Bay, West Antarctica. Antarct Sci.

[12] Mukhtar A, Limbeck A (2013). Recent developments in assessment of bio-accessible trace metal fractions in airborne particulate matter: a review. Anal Chim Acta.

[13] Voutsa D, Samara C (2002). Labile and bioaccessible fractions of heavy metals in the airborne particulate matter from urban and industrial areas. Atmos Environ.

[14] Perron MMG, Strzelec M, Gault-Ringold M, Proemse BC, Boyd PW, Bowie AR (2020). Assessment of leaching protocols to determine the solubility of trace metals in aerosols. Talanta..

[15] Sillanpaa M, Saarikoski S, Hillamo R, Pennanen A, Makkonen U, Spolnik Z (2005). Chemical composition, mass size distribution and source analysis of long-range transported wildfire smokes in Helsinki. Sci Total Environ.

[16] Godoy MLDP, Godoy JM, Roldao LA, Soluri DS, Donagemma RA (2009). Coarse and fine aerosol source apportionment in Rio de Janeiro, Brazil. Atmos Environ.

[17] Canepari S, Pietrodangelo A, Perrino C, Astolfi ML, Marzo ML (2009). Enhancement of source traceability of atmospheric PM by elemental chemical fractionation. Atmos Environ.

[18] Gouws K, Coetzee PP (1997). Determination and partitioning of heavy metals in sediments of the Vaal Dam System by sequential extraction. Water SA.

[19] Kyotani T, Iwatsuki M (2002). Characterization of soluble and insoluble components in PM2.5 and PM10 fractions of airborne particulate matter in Kofu city, Japan. Atmos Environ.

[20] Tessier A, Campbell PGC, Bisson M (1979). Sequential extraction procedure for the speciation of particulate trace metals. Anal Chem.

[21] Ure AM, Quevauviller P, Muntau H, Griepink B (1993). Speciation of heavy metals in soils and sediments. An account of the improvement and harmonization of extraction techniques undertaken under the auspices of the BCR of the Commission of the European Communities. Int J Environ Anal Chem.

[22] Swietilik R, Trojanowska M, Strzelecka M, Bocho-Janiszewska A (2015). Fractionation and mobility of Cu, Fe, Mn, Pb and Zn in the road dust retained on noise barriers along expressway - a potential tool for determining the effects of driving conditions on speciation of emitted particulate metals. Environ Pollut.

[23] Sato K, Tamura T, Furuta N (2008). Partitioning between soluble and insoluble fractions for major and trace elements in size-classified airborne particulate matter collected in Tokyo. J Environ Monit.

[24] Karthikeyan S, Joshi UM, Balasubramanian R (2006). Microwave assisted sample preparation for determining water-soluble fraction of trace elements in urban airborne particulate matter: evaluation of bioavailability. Anal Chim Acta.

[25] Canepari S, Cardarelli E, Giuliano A, Pietrodangelo A (2006). Determination of metals, metalloids and non-volatile ions in airborne particulate matter by a new two-step sequential leaching procedure. Part A: experimental design and optimisation. Talanta..

[26] Canepari S, Cardarelli E, Perrino C, Catrambone M, Pietrodangelo A, Strincone M (2006). Two-stage chemical fractionation method for the analysis of elements and non-volatile inorganic ions in PM10 samples: application to ambient samples collected in Rome (Italy). Atmos Environ.

[27] Canepari S, Cardarelli E, Pietrodangelo A, Strincone M (2006). Determination of metals, metalloids and non-volatile ions in airborne particulate matter by a new two-step sequential leaching procedure. Part B: validation on equivalent real samples. Talanta..

[28] Canepari S, Perrino C, Olivieri F, Astolfi ML (2008). Characterisation of the traffic sources of PM through size-segregated sampling, sequential leaching and ICP analysis. Atmos Environ.

[29] Baffi F, Ianni C, Ravera M, Soggia F, Magi E (1998). Evaluation of the acetate buffer attack of a sequential extraction scheme for marine particulate metal speciation studies by scanning electron microscopy with energy dispersive X-ray analysis. Anal Chim Acta.

[30] Frache R, Abelmoschi ML, Baffi F, Ianni C, Magi E, Soggia F. Trace metals in particulate and sediments (Antarctica). Environmental Contamination in Antarctica - A challenge to analytical chemistry. 2001.

[31] Heal MR, Hibbs LR, Agius RM, Beverland IJ (2005). Total and water-soluble trace metal content of urban background PM10, PM2.5 and black smoke in Edinburgh, UK. Atmos Environ.

[32] Limbeck A, Wagner C, Lendl B, Mukhtar A (2012). Determination of water soluble trace metals in airborne particulate matter using a dynamic extraction procedure with on-line inductively coupled plasma optical emission spectrometric detection. Anal Chim Acta.

[33] Udisti R, Dayan U, Becagli S, Busetto M, Frosini D, Legrand M (2012). Sea spray aerosol in Central Antarctica. Present atmospheric behaviour and implications for paleoclimatic reconstructions. Atmos Environ.

[34] Wang G, Huang L, Gao S, Gao S, Wang L (2002). Characterization of water-soluble species of PM10 and PM2.5 aerosols in urban area in Nanjing, China. Atmos Environ.

[35] Illuminati S, Annibaldi A, Truzzi C, Libani G, Mantini C, Scarponi G (2015). Determination of water-soluble, acid-extractable and inert fractions of cd, Pb and Cu in Antarctic aerosol by square wave anodic stripping voltammetry after sequential extraction and microwave digestion. J Electroanal Chem.

[36] Annibaldi A, Truzzi C, Illuminati S, Bassotti E, Scarponi G (2007). Determination of water-soluble and insoluble (dilute-HCl-extractable) fractions of Cd, Pb and Cu in Antarctic aerosol by square wave anodic stripping voltammetry: distribution and summer seasonal evolution at Terra Nova Bay (Victoria Land). Anal Bioanal Chem.

[37] Fernández Espinosa AJ, Ternero Rodrìguez M (2002). Barragàn de la Rosa FJ, Jiménez Sànchez JC. A chemical speciation of trace metals for fine urban particle. Atmos Environ.

[38] Li H, Wang J, Wang QG, Qian X, Qian Y, Yang M (2015). Chemical fractionation of arsenic and heavy metals in fine particle matter and its implications for risk assessment: a case study in Nanjing, China. Atmos Environ.

[39] Li H, Wang Q, Shao M, Wang J, Wang C, Sun Y (2015). Fractionation of airborne particulate-bound elements in haze-fog episode and associated health risks in a megacity of southeast China. Environ Pollut.

[40] Dabek-Zlotorzynska E, Kelly M, Chen H, Chakrabarti CL (2003). Evaluation of capillary electrophoresis combined with a BCR sequential extraction for determining distribution of Fe, Zn, Cu, Mn, and Cd in airborne particulate matter. Anal Chim Acta.

[41] Sun Y, Hu X, Wu J, Lian H, Chen Y (2014). Fractionation and health risks of atmospheric particle-bound As and heavy metals in summer and winter. Sci Total Environ.

[42] Graney JR, Landis MS, Norris GA (2004). Concentrations and solubility of metals from indoor and personal exposure PM2.5 samples. Atmos Environ.

[43] Drava G, Leardi R, Portesani A, Sales E (1996). Application of chemometrics to the production of friction materials: analysis of previous data and search of new formulations. Chemometr Intell Lab.

[44] Becagli S, Sferlazzo DM, Pace G, di Sarra A, Bommarito C, Calzolai G (2012). Evidence for heavy fuel oil combustion aerosols from chemical analyses at the island of Lampedusa: a possible large role of ships emissions in the Mediterranean. Atmos Chem Phys.

[45] Fernàndez AJ, Ternero M, Barragàn FJ, Jiménez JC (2000). An approach to characterization of sources of urban airborne particles through heavy metal speciation. Chemosphere..

[46] Becagli S, Anello F, Bommarito C, Cassola F, Calzolai G, Di Iorio T (2017). Constraining the ship contribution to the aerosol of the Central Mediterranean. Atmos Chem Phys.

[47] Quevauviller P, Rauret G, López-Sánchez JF, Rubio R, Ure A, Muntau H. The certification of the EDTA-extractable contents (mass fractions) of Cd, Cr, Ni, Pb and Zn in sediment following a three-step sequential extraction procedure. European Commission BCR information reference materials; 1997.

[48] Andrews RN, Keane M, Hanley KW, Feng HA, Ashley K (2015). Manganese speciation of laboratory-generated welding fumes. Anal Methods: Adv Methods Appl.

[49] Nóbrega JA, Pirola C, Richter RC. Think blank. Clean chemistry tools for atomic spectroscopy: Ikonos; 2015.

[50] Bazzano A, Soggia F, Grotti M (2015). Source identification of atmospheric particle-bound metals at Terra Nova Bay Antarctica. Environ Chem.

[51] Bazzano A, Ardini F, Becagli S, Traversi R, Udisti R, Cappelletti D (2015). Source assessment of atmospheric lead measured at Ny-Ålesund, Svalbard. Atmos Environ.

[52] Bazzano A, Cappelletti D, Udisti R, Grotti M (2016). Long-range transport of atmospheric lead reaching Ny-Ålesund: inter-annual and seasonal variations of potential source areas. Atmos Environ.

[53] Ebrahimi-Najafabadi H, Leardi R, Jalali-Heravi M (2014). Experimental design in analytical chemistry—part II: applications. J AOAC Int.

[54] Ebrahimi-Najafabadi H, Leardi R, Jalali-Heravi M (2014). Experimental design in analytical chemistry—part I: theory. J AOAC Int.

[55] Leardi R (2009). Experimental design in chemistry: a tutorial. Anal Chim Acta.

[56] Ciğeroğlu Z, Aras Ö, Pinto CA, Bayramoglu M, Kırbaşlar İ, Lorenzo JM (2018). Optimization of ultrasound-assisted extraction of phenolic compounds from grapefruit (Citrus paradisi Macf.) leaves via D-optimal design and artificial neural network design with categorical and quantitative variables. J Sci Food Agric.

[57] Leardi R, Melzi C, Polotti G. CAT (Chemometric Agile Tool), freely downloadable from http://gruppochemiometria.it/index.php/software. 2019. Accessed 20 Mar 2020.

[58] Wedepohl KH (1995). The composition of the continental crust. Geochim Cosmochim Acta.

[59] Goldberg ED, Riley JP, Skirrow G (1965). Chapter 5: minor elements in sea water. Chemical oceanography.

[60] Mann HB, Whitney DR (1947). On a test of whether one of two random variables is stochastically larger than the other. Ann Math Stat.

[61] Sheppard PR, Helsel DR, Speakman RJ, Ridenour G, Witten ML (2012). Additional analysis of dendrochemical data of Fallon, Nevada. Chem-Bio Inter.

[62] Maher WA, Krikowa F, Foster SD, Ellwood MJ, Bennett WW (2018). Antimony measurements in environmental matrices: seven considerations. J Anal Atom Spectr.

[63] Filgueiras AV, Lavilla I, Bendicho C (2002). Chemical sequential extraction for metal partitioning in environmental solid samples. J Environ Monit.

[64] Van Elteren JT (2004). Novel approaches for determination of the chemical availability of metal(loid)s in soil based on the kd concept. Acta Chim Slov.

[65] Allison JD, Allison TL. Partition coefficients for metals in surface water, soil, and waste. U.S. Environmental Protection Agency; 2005.

[66] Henderson P, Henderson GM (2009). The Cambridge handbook of earth science data.

[67] Moreno T, Querol X, Alastuey A, Gibbons W (2008). Identification of FCC refinery atmospheric pollution events using lanthanoid- and vanadium-bearing aerosols. Atmos Environ.

[68] Moreno T, Querol X, Alastuey A, Pey J, Minguillón MC, Pérez N (2008). Lanthanoid geochemistry of urban atmospheric particulate matter. Environ Sci Technol.

[69] Kryc KA, Murray RW, Murray DW (2003). Al-to-oxide and Ti-to-organic linkages in biogenic sediment: relationships to paleo-export production and bulk Al/Ti. Earth Planet Sci Lett.

