# Supplementary material for: Optimization of a sequential extraction procedure for trace elements in Arctic PM10
Source: Anal Bioanal Chem. 2020 Aug 20;412(27):7429–40. doi: 10.1007/s00216-020-02874-4 (PMC7533259; doi:10.1007/s00216-020-02874-4)
Supplement: Supplementary file 1 — (PDF 469 kb) [file 216_2020_2874_MOESM1_ESM.pdf]

Analytical and Bioanalytical Chemistry

Electronic Supplementary Material

Optimization of a sequential extraction procedure for trace elements in Arctic PM_{10}

Eleonora Conca, Mery Malandrino, Agnese Giacomino, Emanuele Costa,
Francisco Ardini, Paolo Inaudi, Ornella Abollino

Table S1 Operating conditions applied for the instrumental analysis

ICP-OES		
**Analyte**	**λ (nm)**	**Torch position**
Al	396.2	Axial - Radial
Ca	317.9	Radial
Fe	238.2	Axial - Radial
K	766.5	Axial - Radial
Mg	285.2	Axial - Radial
Na	589.6	Axial
Pb	220.4	Axial - Radial
Si	251.6	Axial - Radial
Ti	334.9	Axial
Zn	206.2	Axial - Radial

HR-ICP-MS		
**Analyte**	**Isotopes**	**Resolution**
As	75	Medium
Ba	135-137	Medium
Cd	111-112-114	Low - Medium
Co	59	Medium
Cr	52-53	Medium
Cu	63-65	Medium
Fe	56-57	Medium
Mn	55	Medium
Ni	60-61-62	Medium
Pb	206-207-208	Medium
Sb	121-123	Medium
Ti	47-49	Medium
V	51	Medium

Table S2 Crustal (CEF) and marine (MEF) enrichment factors calculated for the CRM NIST 1648a

	**CEF**	**MEF**
Al	1.00	8490000
As	130	95300
Ca	4.48	362
Cd	1630	1660000
Co	3.49	444000
Cr	25.9	19900000
Cu	96.3	504000
Fe	2.87	9710000
K	0.832	68.8
Mg	1.36	14.9
Mn	3.38	978000
Na	0.373	1.00
Ni	9.84	100000
Pb	870	541000000
Sb	331	225000
Si	0.952	106000
Ti	2.91	9960000
V	5.41	157000
Zn	208	1190000

$$CEF_i = \frac{C_{i\,PM}/C_{r\,PM}}{C_{i\,crust^*}/C_{r\,crust^*}}$$

$$MEF_i = \frac{C_{i\,PM}/C_{r\,PM}}{C_{i\,sea^+}/C_{r\,sea^+}}$$

* Wedepohl (1995)

⁺ Goldberg (1965)

Table S3 Recovery percentages obtained for the CRM NIST 1648a with the ten tested procedures

	HPW - Stir	Buffer - Stir	CH_3COOH - Stir	HCl - Stir	HNO_3 - Stir	HPW - US	Buffer - US	CH_3COOH - US	HCl - US	HNO_3 - US
**Al**	55 ± 3	61 ± 3	56 ± 7	56 ± 5	62 ± 3	59 ± 2	57 ± 2	51 ± 4	53 ± 3	48 ± 4
**As**	109 ± 3	107 ± 8	108 ± 3	105 ± 3	105 ± 4	112 ± 6	105 ± 2	99 ± 3	95 ± 6	100 ± 2
**Ba**	77 ± 1	83 ± 6	78 ± 3	82 ± 3	78 ± 3	80.7 ± 0.5	78 ± 2	71 ± 2	69 ± 2	69 ± 3
**Ca**	108 ± 2	106 ± 4	102 ± 2	106 ± 2	105 ± 2	109 ± 2	102 ± 2	105 ± 3	104 ± 5	103 ± 2
**Cd**	101 ± 5	103 ± 3	100 ± 1	105 ± 3	101 ± 4	103 ± 5	100 ± 4	97 ± 1	96 ± 4	100 ± 7
**Co**	76 ± 3	81 ± 2	78 ± 4	81 ± 2	81 ± 3	84 ± 4	76 ± 3	74.4 ± 0.5	70 ± 2	74 ± 4
**Cr**	32 ± 3	36 ± 3	32 ± 4	34 ± 2	35 ± 3	34 ± 2	35 ± 1	31 ± 1	30 ± 2	31 ± 3
**Cu**	84 ± 3	91 ± 4	89 ± 2	95 ± 4	97 ± 2	88 ± 5	84.5 ± 0.7	85 ± 3	88 ± 4	91 ± 5
**Fe**	83 ± 1	92 ± 7	83 ± 4	84 ± 3	112 ± 6	90 ± 1	80 ± 3	78 ± 4	98 ± 3	71 ± 2
**K**	56 ± 2	57 ± 3	54 ± 6	63 ± 4	70 ± 6	57 ± 2	54 ± 2	50 ± 4	57 ± 3	56 ± 5
**Mg**	87.1 ± 0.7	90 ± 3	86 ± 3	88 ± 1	89 ± 2	91 ± 2	84 ± 2	83 ± 3	83 ± 2	80.6 ± 0.9
**Mn**	92 ± 1	95 ± 5	93.3 ± 0.6	96 ± 2	101 ± 2	95 ± 2	94 ± 2	86 ± 2	87 ± 5	91 ± 2
**Na**	64 ± 1	64 ± 2	69 ± 4	57.0 ± 0.8	59 ± 1	64.8 ± 0.8	69 ± 3	63 ± 1	59 ± 2	57 ± 3
**Ni**	93 ± 2	98 ± 3	93 ± 4	95 ± 4	97 ± 1	101 ± 3	92 ± 2	86 ± 3	92 ± 3	90 ± 3
**Pb**	97 ± 2	100 ± 5	96 ± 4	102 ± 1	102 ± 4	104 ± 4	94 ± 2	92 ± 2	96 ± 1	97 ± 1
**Sb**	65 ± 2	64 ± 3	67 ± 2	63 ± 2	64 ± 2	66 ± 2	62 ± 1	58.0 ± 0.1	57 ± 2	54 ± 1
**Si**	13 ± 3	14 ± 4	13 ± 5	13 ± 3	17 ± 4	14 ± 5	14 ± 2	12 ± 3	14 ± 4	11 ± 3
**Ti**	29.5 ± 0.7	32.6 ± 0.5	31 ± 3	33 ± 2	37 ± 1	30 ± 2	32 ± 1	27 ± 1	31 ± 1	28 ± 2
**V**	86 ± 1	90 ± 5	84 ± 4	88.1 ± 0.8	91 ± 1	90 ± 3	84 ± 1	81 ± 3	80 ± 2	84 ± 4
**Zn**	96 ± 2	99 ± 4	97 ± 2	99 ± 2	102 ± 1	101 ± 3	98 ± 3	93 ± 2	96 ± 3	94 ± 3

Stir = 16 hours stirring; US = 15 min ultrasounds; Buffer = CH_3COOH/CH_3COONH_4 buffer.

Table S4 Extraction percentages obtained for the CRM BCR 701 with the five tested procedures: a) fraction I; b) fraction II

a)	FRACTION I				
	**HPW - Stir**	**Buffer - Stir**	**CH_3COOH - Stir**	**HCl - Stir**	**HNO_3 - Stir**
**Cd**	1.03 ± 0.06	56 ± 2	80 ± 2	83 ± 2	86.6 ± 0.7
**Cr**	0.42 ± 0.04	0.75 ± 0.01	4.36 ± 0.03	10.6 ± 0.2	14.1 ± 0.5
**Cu**	2.3 ± 0.2	14 ± 1	49.4 ± 0.9	60.8 ± 0.4	64 ± 3
**Ni**	5 ± 1	15 ± 1	23.5 ± 0.6	33 ± 3	33 ± 1
**Pb**	0.32 ± 0.03	0.76 ± 0.08	28.6 ± 0.3	71 ± 5	77 ± 7
**Zn**	16 ± 5	41.6 ± 0.4	70 ± 10	66 ± 3	62.0 ± 0.5

b)	FRACTION II				
	**HPW - Stir**	**Buffer - Stir**	**CH_3COOH - Stir**	**HCl - Stir**	**HNO_3 - Stir**
**Cd**	94 ± 5	42.8 ± 0.3	15 ± 1	8.0 ± 0.4	8.1 ± 0.3
**Cr**	104 ± 3	105 ± 3	98 ± 3	90 ± 4	89 ± 1
**Cu**	80 ± 4	73 ± 2	42 ± 2	29.0 ± 0.2	27.3 ± 0.3
**Ni**	88 ± 4	82.0 ± 0.1	74 ± 3	65 ± 4	60 ± 2
**Pb**	75 ± 4	73 ± 1	49 ± 1	22 ± 3	18 ± 2
**Zn**	98 ± 5	72 ± 5	56 ± 4	49 ± 3	44 ± 2

Stir = 16 hours stirring; Buffer = CH_3COOH/CH_3COONH_4 buffer.

Table S5 Extraction percentages (fraction I) calculated for the ten PM_{10} samples collected in Ny-Ålesund in 2010 and 2012

	23Mar10	27Mar10	17Apr12	25Apr12	29Apr12	26Jun10	9Jul10	18Jul12	22Jul12	26Jul12
**Al**	6.9 ± 0.3	5.7 ± 0.3	n.a.	22.5 ± 0.5	23.4 ± 0.6	5.1 ± 0.2	3.21 ± 0.01	7.7 ± 0.4	9.60 ± 0.02	6.5 ± 0.2
**As**	79 ± 3	57 ± 1	95 ± 3	94 ± 4	95 ± 6	47 ± 2	69 ± 4	79 ± 5	95 ± 2	88 ± 5
**Ba**	46 ± 2	33 ± 1	72 ± 2	64 ± 3	74 ± 3	22 ± 1	20 ± 1	26 ± 1	45 ± 3	38 ± 2
**Ca**	100 ± 2	94 ± 2	100 ± 4	78.3 ± 0.4	100 ± 4	65.0 ± 0.9	60 ± 3	64 ± 2	97 ± 4	100 ± 2
**Cd**	95 ± 5	32 ± 1	93 ± 6	79 ± 4	89 ± 4	100 ± 7	< PB	< PB	77 ± 8	76 ± 4
**Co**	67 ± 2	57 ± 4	65 ± 3	64 ± 5	78 ± 3	57 ± 1	62 ± 7	59 ± 2	83 ± 4	68 ± 3
**Cr**	15.1 ± 0.6	8.5 ± 0.2	19.8 ± 0.8	20.4 ± 0.5	22.2 ± 0.4	20.1 ± 0.6	11.6 ± 0.5	9.0 ± 0.4	52 ± 2	9.0 ± 0.4
**Cu**	81 ± 2	46 ± 2	91 ± 2	63 ± 2	84 ± 2	57 ± 2	85 ± 2	60 ± 2	56 ± 1	93 ± 2
**Fe**	15.8 ± 0.5	10.9 ± 0.5	33.8 ± 0.5	33.7 ± 0.6	41.8 ± 0.5	11.7 ± 0.5	9.5 ± 0.2	16.6 ± 0.6	47 ± 2	17.5 ± 0.3
**K**	77 ± 2	63 ± 3	84 ± 2	86 ± 2	93 ± 3	79 ± 3	52 ± 1	64 ± 4	92 ± 2	57 ± 1
**Mg**	29.4 ± 0.5	23.3 ± 0.2	97 ± 4	95 ± 2	98 ± 3	16.9 ± 0.5	12.0 ± 0.2	85 ± 4	97 ± 1	91 ± 2
**Mn**	51 ± 2	36.7 ± 0.9	81 ± 1	76.6 ± 0.3	82 ± 3	19.0 ± 0.4	24 ± 2	50 ± 2	78 ± 2	68 ± 1
**Na**	67 ± 1	69.9 ± 0.6	99 ± 2	98 ± 4	99 ± 2	80.0 ± 0.7	85 ± 1	97 ± 3	100 ± 3	98 ± 3
**Ni**	61 ± 3	78 ± 5	84 ± 1	58 ± 1	91 ± 2	41 ± 1	98 ± 2	36.5 ± 0.8	86 ± 2	75 ± 2
**Pb**	97 ± 3	92 ± 4	99 ± 3	77 ± 2	91 ± 4	57 ± 3	53 ± 2	63 ± 2	70 ± 3	78 ± 3
**Sb**	66 ± 2	15.6 ± 0.8	74 ± 4	67 ± 4	62 ± 3	< PB	< PB	34 ± 2	46 ± 1	30 ± 2
**Si**	< PB	< PB	12.5 ± 0.5	18.7 ± 0.6	19.5 ± 0.7	< PB	72 ± 4	11.1 ± 0.8	24.9 ± 0.3	16.2 ± 0.4
**Ti**	4.8 ± 0.1	2.1 ± 0.1	11.3 ± 0.5	29 ± 2	39 ± 1	3.6 ± 0.2	2.8 ± 0.1	3.3 ± 0.2	10.1 ± 0.8	2.3 ± 0.1
**V**	80 ± 5	60 ± 2	68 ± 2	77 ± 4	71 ± 2	73 ± 3	90 ± 5	71 ± 3	97.3 ± 0.6	89 ± 3
**Zn**	100 ± 1	79.8 ± 0.7	95 ± 2	< PB	< PB	100.0 ± 0.8	100 ± 1	< PB	89 ± 2	72 ± 1

PB = procedural blank

Table S6 Enrichment factors calculated for the Arctic PM_{10} samples: a) crustal (CEF); b) marine (MEF)

a)	23Mar10	27Mar10	17Apr12	25Apr12	29Apr12	26Jun10	9Jul10	18Jul12	22Jul12	26Jul12
**Al**	1.00	1.00	1.00	1.00	1.00	1.00	1.00	1.00	1.00	1.00
**As**	973	204	193	143	138	16.1	184	58.4	76.6	63.4
**Ba**	2.17	1.85	1.25	1.41	1.64	0.863	1.33	0.87	1.34	1.38
**Ca**	8.80	5.66	5.76	7.08	10.34	6.16	9.69	6.56	9.47	7.30
**Cd**	4960	1770	406	386	375	756	752	24.6	202	110
**Co**	3.31	4.35	2.20	1.28	2.12	3.47	-	10.3	8.21	5.49
**Cu**	58	8.95	59.6	36.7	59.9	7.47	0.447	34.8	68.7	147
**Fe**	4.32	3.00	2.26	2.47	2.27	2.63	3.40	2.67	2.46	2.59
**K**	4.67	2.74	3.55	4.97	7.57	3.68	3.04	3.27	5.11	2.17
**Mg**	-	-	23.0	31.5	52.5	-	-	20.5	34.9	13.8
**Mn**	7.88	4.77	3.53	3.65	3.78	2.33	3.30	3.03	2.92	3.00
**Na**	47.0	37.1	65.5	103	162	67.3	113	71.1	135	34.1
**Ni**	60.7	47.9	33.1	25.7	29.2	41.1	175	242	187	51.2
**Pb**	550	167	79.4	76.1	67.1	5.87	15.1	1.92	-	-
**Ti**	0.975	0.855	0.962	1.08	0.939	1.15	1.18	1.19	0.905	0.909
**V**	13.4	6.00	5.21	5.96	5.45	16.1	82.3	17.2	155	36.6
**Zn**	390	116	86.5	70.7	62.6	49.4	216	54.3	42.3	72.8

b)	23Mar10	27Mar10	17Apr12	25Apr12	29Apr12	26Jun10	9Jul10	18Jul12	22Jul12	26Jul12
**Al**	67400	85400	48300	30800	19600	47100	28100	44500	23500	92800
**As**	5640	1500	804	379	232	65.0	444	224	155	507
**Ba**	420	455	174	125	92.4	117	107	111	90.2	368
**Ca**	5.64	4.60	2.65	2.07	1.93	2.76	2.59	2.78	2.11	6.44
**Cd**	40000	18100	2350	1420	881	4260	2530	131	566	1220
**Co**	3350	5560	1590	590	622	2450	-	6880	2890	7640
**Cu**	2420	471	1780	695	723	216	7.72	953	993	8390
**Fe**	116000	102000	43600	30300	17800	49400	38100	47400	23000	95800
**K**	3.06	2.27	1.67	1.49	1.45	1.69	0.831	1.42	1.17	1.96
**Mg**	-	-	1.44	1.25	1.33	-	-	1.18	1.06	1.66
**Mn**	18100	13900	5800	3820	2520	3730	3160	4590	2330	9470
**Na**	1.00	1.00	1.00	1.00	1.00	1.00	1.00	1.00	1.00	1.00
**Ni**	4910	4910	1920	950	686	2320	5910	12900	5260	5710
**Pb**	2710000	1040000	281000	171000	96200	20200	30900	6250	-	-
**Ti**	26500	29400	18700	13300	7410	21900	13400	21300	8550	34000
**V**	3090	1750	862	628	365	2590	7910	2620	12400	11600
**Zn**	17600	6660	2810	1460	824	1560	4070	1620	667	4540

Table S7 Total concentrations obtained by direct digestion for the ten PM_{10} samples collected in Ny-Ålesund in 2010 and 2012 (Bazzano et al., 2016, Conca et al., 2019)

	23Mar10	27Mar10	17Apr12	25Apr12	29Apr12	26Jun10	9Jul10	18Jul12	22Jul12	26Jul12
**Al** (ng/m^3)	5.26 ± 0.05	11.9 ± 0.2	9.6 ± 0.3	6.4 ± 0.2	7.4 ± 0.4	9.43 ± 0.09	3.87 ± 0.04	6.8 ± 0.1	5.5 ± 0.1	7.1 ± 0.3
**As** (pg/m^3)	132 ± 1	62.8 ± 0.6	48 ± 3	24 ± 2	26 ± 3	3.91 ± 0.04	18.3 ± 0.2	10.3 ± 0.9	10.9 ± 0.9	11.6 ± 0.5
**Ba** (pg/m^3)	98 ± 1	190 ± 2	104 ± 4	78 ± 3	105 ± 4	70.2 ± 0.7	44.3 ± 0.4	51 ± 1	64 ± 2	84 ± 2
**Ca** (ng/m^3)	17.6 ± 0.2	25.7 ± 0.3	21.1 ± 0.2	17.3 ± 0.1	29.2 ± 0.2	22.1 ± 0.2	14.2 ± 0.1	17.0 ± 0.2	19.9 ± 0.4	19.6 ± 0.3
**Cd** (pg/m^3)	34.4 ± 0.3	27.8 ± 0.3	5.1 ± 0.8	3.3 ± 0.7	3.7 ± 0.4	9.39 ± 0.09	3.83 ± 0.04	0.22 ± 0.04	1.5 ± 0.2	1.0 ± 0.4
**Co** (pg/m^3)	2.61 ± 0.03	7.75 ± 0.08	3.2 ± 0.2	1.23 ± 0.02	2.36 ± 0.08	4.90 ± 0.05	< 0.052	10.5 ± 0.4	6.8 ± 0.3	5.8 ± 0.3
**Cu** (pg/m^3)	56.6 ± 0.6	19.7 ± 0.2	106 ± 2	43 ± 2	82 ± 2	13.0 ± 0.1	0.32 ± 0.01	44 ± 1	70 ± 2	192 ± 3
**Fe** (ng/m^3)	9.06 ± 0.09	14.3 ± 0.1	8.7 ± 0.2	6.32 ± 0.08	6.74 ± 0.07	9.90 ± 0.09	5.25 ± 0.05	7.3 ± 0.2	5.4 ± 0.2	7.29 ± 0.08
**K** (ng/m^3)	9.08 ± 0.09	12.1 ± 0.1	12.6 ± 0.1	11.79 ± 0.03	20.8 ± 0.2	12.9 ± 0.1	4.35 ± 0.04	8.25 ± 0.07	10.4 ± 0.1	5.7 ± 0.1
**Mg** (ng/m^3)	n.a.	n.a.	38 ± 1	35.2 ± 0.3	68.1 ± 0.9	n.a.	n.a.	24.4 ± 0.5	33.6 ± 0.7	17.0 ± 0.4
**Mn** (pg/m^3)	282 ± 3	387 ± 4	231 ± 5	159 ± 5	191 ± 8	149 ± 1	86.9 ± 0.9	141 ± 2	110 ± 2	144 ± 1
**Na** (ng/m^3)	82.0 ± 0.8	146 ± 1	210 ± 20	220 ± 10	398 ± 5	210 ± 2	145 ± 1	161 ± 4	250 ± 20	80 ± 4
**Ni** (pg/m^3)	76.7 ± 0.8	137 ± 1	77 ± 3	40 ± 3	52 ± 2	93.0 ± 0.9	163 ± 2	396 ± 7	247 ± 9	87 ± 3
**Pb** (pg/m^3)	635 ± 6	437 ± 4	168 ± 6	107 ± 2	109 ± 3	12.1 ± 0.1	12.8 ± 0.1	2.87 ± 0.06	< 0.032	< 0.032
**Ti** (pg/m^3)	207 ± 2	410 ± 4	370 ± 10	278 ± 8	280 ± 10	438 ± 4	184 ± 2	327 ± 5	200 ± 10	259 ± 9
**V** (pg/m^3)	48.2 ± 0.5	48.9 ± 0.5	34 ± 2	26.2 ± 0.8	28 ± 1	104 ± 1	218 ± 2	80.3 ± 0.5	580 ± 20	177 ± 9
**Zn** (pg/m^3)	1380 ± 10	929 ± 9	560 ± 20	300 ± 20	310 ± 10	313 ± 3	561 ± 6	250 ± 10	157 ± 9	350 ± 20

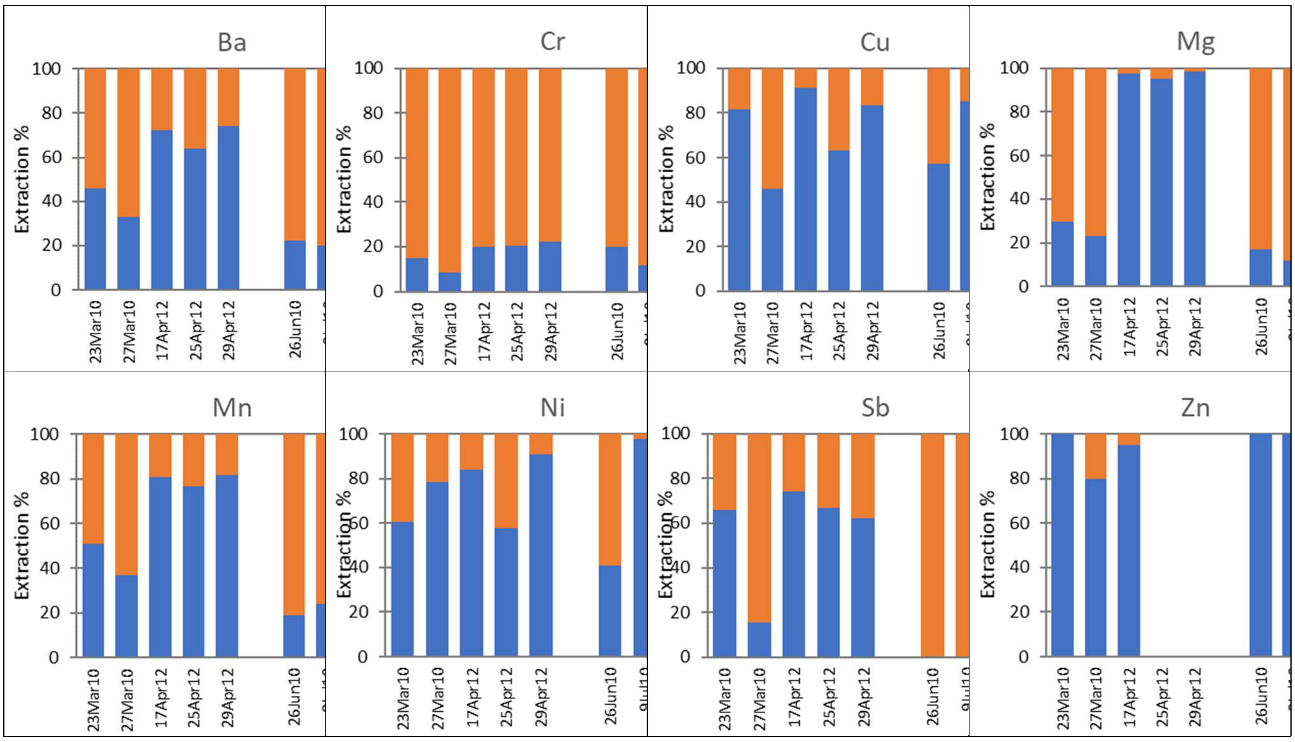

Fig. S1 Extraction percentages obtained for the ten Arctic PM_{10} samples

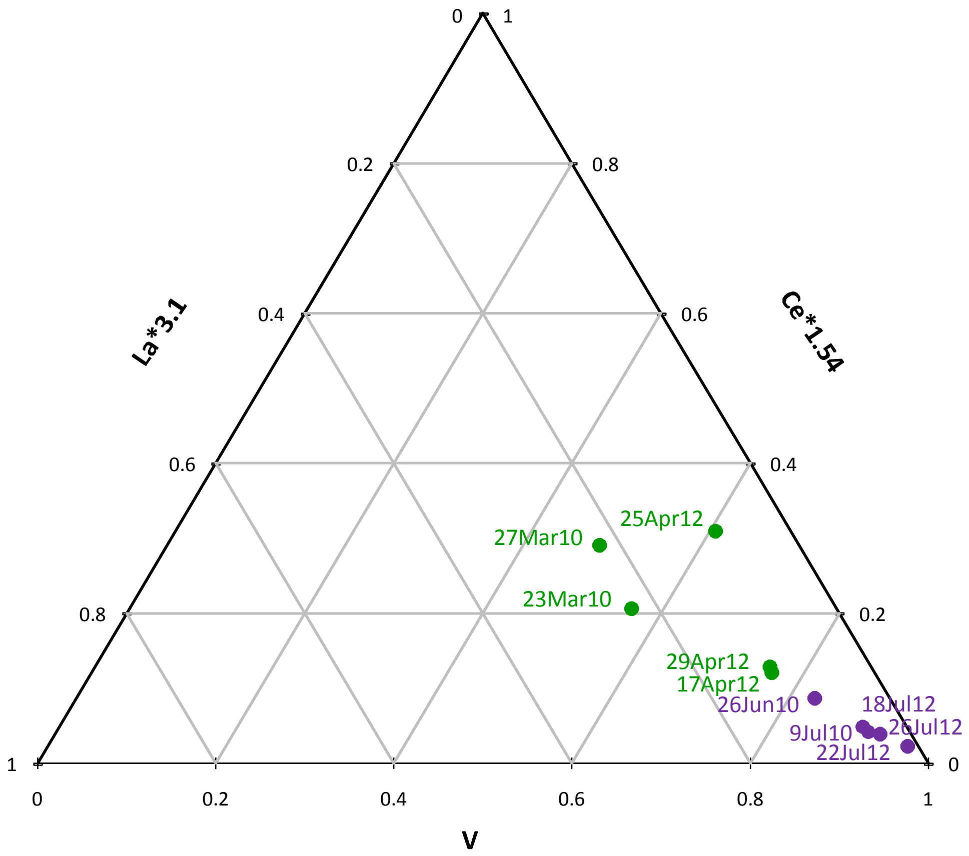

Fig. S2 Ce-La-V ternary diagram for the investigated 2010 and 2012 Arctic PM_{10} samples

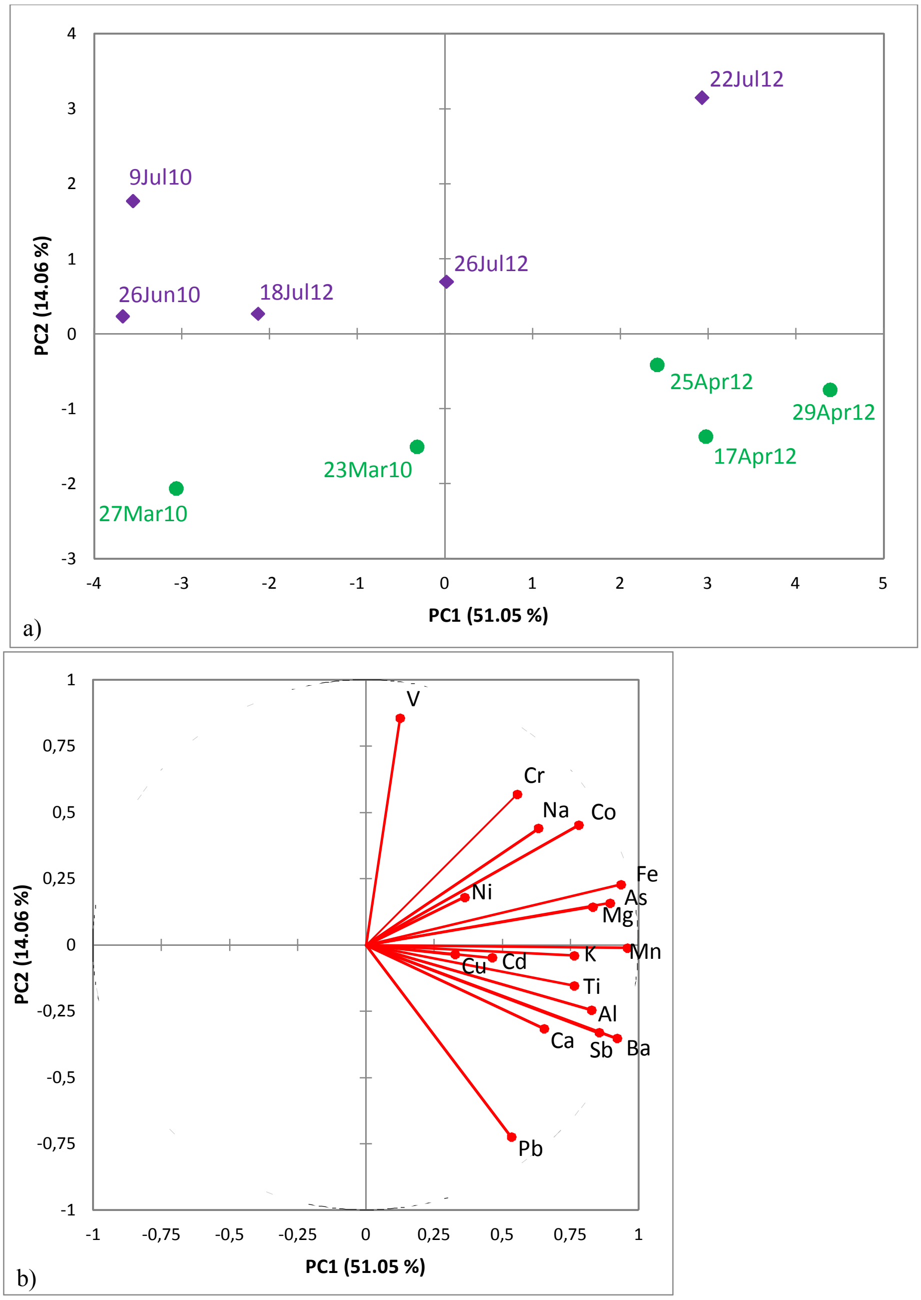

Fig. S3 Principal Component Analysis (PC1 vs. PC2) for the results of the sequential extraction procedure (fraction I) performed on ten PM_{10} samples collected in Ny-Ålesund in 2010 and 2012: a) score plot; b) loading plot
